# Introducing a New Investigation Sheet at Almanagil Teaching Hospital in Sudan: A Quality Improvement Project

**DOI:** 10.7759/cureus.92078

**Published:** 2025-09-11

**Authors:** Abubakr Muhammed, Abdalmahmoud Asadig Kanan Ahmed, Ammar Ali Elmubark Hamid, Marwa Yousif, Abdelrahman Sahnon Abaker Sahnon, Mohammed Abdalazeem Alsheikh Ahmed, Mohamed Alaeldin Mohamed Gasim, Ithar Musa Mohamed Ali Abdalla, Alaa Qassim Ali Abdalla, Mohamed Bakheit Mohamed Bakheit, Ola Atif Mubarak Abdullah, Mohanned Esameldin Olish Mohamed, Mohammed Osman, Eman Esam Hassan Ali, Lena Abdelmoneim Elhadi Elamin, Ahmed Yahia Mahadi Babiker, Mohamedelmugtaba Awad Mohamed Abuzaid, Alshyma Alfatih Mohamed Abdallah, Ijlal Eltaiyb Ali Mohammed, Moiez Mohammed Aboudi Ahmed

**Affiliations:** 1 Surgery, University of Gezira, Wad Madani, SDN; 2 General Surgery, Almanagil Teaching Hospital, Almanagil, SDN; 3 General Surgery, Abu Dhabi Department of Health, Abu Dhabi, ARE; 4 General Practice, Medical Council of Ireland, Dublin, IRL; 5 General Surgery, Royal College of Surgeons of Edinburgh, Edinburgh, GBR; 6 General Surgery, University of Medical Sciences and Technology (UMST), Khartoum, SDN

**Keywords:** clinical audit, documentation, quality improvement project, standardized investigation sheet, training

## Abstract

Background: Incomplete medical documentation threatens patient safety and efficiency. At Almanagil Teaching Hospital (Sudan), baseline audits revealed only 21% completeness of investigation forms.

Aim: This study aimed to increase documentation completeness from 21% to ≥90% within six weeks through a standardized investigation sheet and staff training.

Methods: A prospective observational quality improvement project (May-June 2025) applied two Plan-Do-Study-Act (PDSA) cycles. Cycle 1 audited 50 forms to assess baseline practice. Interventions included a redesigned standardized sheet, educational workshops, laminated samples, and posters. Cycle 2 audited 51 forms to assess improvement and gather feedback. Completeness was defined as the proportion of eligible fields documented. Two auditors independently reviewed 20% of forms (κ = 0.89). Because samples were independent, Pearson χ² and two-proportion z-tests were used, with Holm-Bonferroni correction for multiple comparisons. Effect sizes were reported with 95% confidence intervals. Run charts tracked weekly progress. Balancing measures included form completion time and laboratory turnaround.

Results: Mean completeness increased from 21.7% (95% CI: 14.5-28.9) to 94.2% (95% CI: 91.1-97.3) (p < 0.001). Improvements were seen across patient identifiers (0-40% → 100%) and critical labs (12-14% → ≥98%). Median form completion time increased slightly (3.2 → 3.7 minutes; p = 0.12), with no change in laboratory turnaround (2.0 → 2.1 hours; p = 0.64). Staff feedback informed refinements, including urgent flags and color-coded fields.

Conclusions: A standardized sheet with training and audit cycles significantly improved documentation completeness. Sustainability measures include weekly audits, documentation champions, rejection of incomplete forms, quarterly refresher training, and electronic medical record integration.

## Introduction

High-quality medical record keeping is fundamental to patient safety, effective clinical care, and robust health system management [1]. Accurate and reliable medical records serve as the primary source of clinical information, ensuring that healthcare providers can make informed decisions throughout the patient's continuum of care. The clarity, accuracy, and timeliness of documentation not only facilitate multidisciplinary communication but also directly impact patient outcomes, as well-documented records reduce the risk of diagnostic or treatment errors and support continuity of care [1].

However, many healthcare institutions, especially in low- and middle-income countries, continue to face persistent challenges in achieving comprehensive and standardized documentation practices [2]. Factors such as limited resources, inadequate training, and the absence of standardized templates often contribute to these discrepancies. As a result, incomplete or inconsistent records are commonly observed, undermining the quality of care provided to patients [2].

Incomplete or inconsistent records contribute to care delays, medical errors, and inefficiency across the continuum of care [3]. For instance, if laboratory or imaging investigations are not correctly documented, healthcare teams may miss critical information, resulting in redundant testing, miscommunication, and delays in patient management [3]. Previous quality improvement (QI) studies demonstrate that standardized tools, combined with staff education, can markedly improve documentation completeness and accuracy [2-5].

Recent studies in Sudan and Ethiopia have shown that deficiencies in medical documentation hinder quality care and highlight the need for targeted improvements [2-5].

Rationale

Standardized investigation sheets reduce variability, improve completeness, and allow consistent auditing.

Aim statement

This study aimed to increase the completeness of investigation documentation from 21% to ≥90% within six weeks (May-June 2025) by implementing a standardized investigation sheet and targeted educational sessions for physicians and nurses. Process, outcome, and balancing measures were defined to track improvement, including mean completion rate, field-specific compliance, and staff workflow impact.

Audits of discharge summaries and investigation checklists have revealed significant documentation gaps, which not only affect patient care but also impede institutional performance assessment and research activities [5]. Similarly, investigation of clinical records from tertiary care and specialized hospitals has demonstrated suboptimal compliance with recommended documentation standards, further affirming the need for intervention [6].

Global improvement programs stress that systematic QI in record keeping is essential for better healthcare delivery [7]. Implementing QI initiatives such as standardized forms, regular audits, feedback mechanisms, and training has all demonstrated positive impacts on record completeness and accuracy [7]. Additionally, structured interventions that focus on ongoing education and systematic clinical audits support sustained improvements in documentation standards [8]. For example, QI cycles involving the design and implementation of specialized checklists have been associated with measurable increases in record completeness and adherence to institutional guidelines [8].

Hospitals that implement such quality improvement projects (QIP) report increased adherence to best practices and improved patient outcomes [9]. Structured approaches to discharge documentation and patient follow-up processes not only enhance the quality of care delivered but also enable healthcare providers to reliably track interventions and clinical progress, ultimately contributing to safer patient journeys [9].

On an international level, health authorities such as the World Health Organization (WHO) emphasize the necessity of robust quality frameworks to sustain improvements in documentation and general care delivery processes [10]. These frameworks advocate for the integration of data-driven decision-making, performance monitoring, and ongoing staff engagement in achieving high standards of care [10].

Despite advances in medical record documentation, variability in investigation documentation remains a significant barrier to safe and efficient patient care at Almanagil Teaching Hospital in Sudan. To address this gap, this QIP aimed to increase the completeness of investigation documentation from 21% to 90% within six weeks through the design, implementation, and evaluation of a standardized investigation sheet, coupled with targeted educational sessions for physicians and nurses. We hypothesized that this structured intervention, supported by iterative feedback, would result in measurable improvements in documentation quality, enhance patient safety, and strengthen institutional clinical processes.

## Materials and methods

This QIP was conducted at Almanagil Teaching Hospital, Gezira State, Sudan, to enhance documentation practices for clinical investigations. The intervention spanned two QIP cycles over a total duration of 47 days, from May 1, 2025, to June 16, 2025. A prospective observational design was applied, incorporating structured QI methods aligned with the Standards for Quality Improvement Reporting Excellence (SQUIRE) 2.0, including Plan-Do-Study-Act (PDSA) cycles, process/outcome/balancing measures, and continuous stakeholder engagement.

Context

The project was conducted in the medical and surgical wards of Almanagil Teaching Hospital, a 350-bed tertiary facility in Gezira State, Sudan, serving ~500 outpatients daily. Staffing included 120 physicians, 180 nurses, and 40 laboratory staff. No other concurrent documentation initiatives were in place.

Ethical considerations

This QIP was reviewed and approved by the Institutional Ethics Committee (IEC) of Almanagil Teaching Hospital (approval number: 2024-QIP-003). All data were de-identified. No external funding was received.

Building upon the investigation sheet originally used at Dongola Specialized Hospital, the authors developed a revised version tailored to the needs of Almanagil Teaching Hospital. Prior to implementation, formal approvals were obtained, ensuring alignment with institutional requirements. The new sheet incorporated mandatory fields for patient identifiers, collection dates, sequential test results, and physician authentication. The design emphasized usability, minimized ambiguity, and allowed for easy auditing of completed forms. The modifications were collaboratively refined by the authors, surgical consultants, and relevant stakeholders at Almanagil. Appendices A and B provide a visual representation of the newly introduced investigation sheet, highlighting key updates and formatting adjustments.

First cycle (pre-intervention state and root cause analysis: May 1-15, 2025)

In the initial pre-intervention phase, a prospective audit of 50 randomly selected investigation sheets was carried out. Fragmented documentation was common: essential details such as patient identification, dates, signatures, and most test parameters were inconsistent or rarely recorded. Patient name and gender appeared in only a minority of cases, while entries like age, hospital number, and blood group were frequently missing. The mean completion rate for laboratory and biochemical entries was just 21.7%. Based on these findings, the QIP team performed a structured root cause analysis using staff interviews and discussions with physicians, nurses, and laboratory personnel. Key contributors identified were lack of a standardized investigation sheet, insufficient training, and weak accountability. Process measures (field-specific completion rates), outcome measures (mean overall completion), and balancing measures (impact on staff workflow) were defined to guide the intervention.

Intervention standardization and staff training (May 16-31, 2025 (16 days))

To address documentation gaps, a standardized investigation sheet was developed, drawing on WHO documentation recommendations. The new form required fields for patient and doctor identifiers, collection date and time, sequential test results, and mandatory staff signatures. Educational workshops underscored the legal and clinical necessity of accurate documentation. Laminated sample forms and instructional posters were distributed throughout clinical and laboratory spaces to reinforce best practices. Training sessions included hands-on exercises, simulated audits, and guidance on prioritizing urgent tests. Staff were encouraged to provide feedback for iterative refinement.

Second cycle (post-intervention audit and process refinement: June 1-15, 2025 (15 days))

Following training and form implementation, a post-intervention audit was conducted, reviewing 51 investigation sheets. These forms were evaluated for completeness, and results were directly compared with pre-intervention findings to assess improvement. Staff feedback on the revised sheet was actively gathered to evaluate usability and persistent barriers. Run charts were created to visualize changes in documentation completeness over time, and statistical analysis using McNemar's test assessed the significance of improvements.

Based on this feedback, further refinements were made to the investigation sheet. The layout was simplified for frequently ordered tests, an "URGENT" field was added for time-sensitive cases, and color-coding was implemented to highlight critical values. Supporting measures included instructing laboratory staff to reject incomplete forms, appointing documentation champions to monitor compliance, and scheduling weekly review meetings to sustain improvements.

Data analysis

All investigation forms from both cycles were assessed according to predefined criteria for completeness, accuracy, and adherence to documentation standards. Quantitative analysis focused on parameter-specific completion rates before and after the intervention, while qualitative feedback was thematically analyzed to guide process refinements.

During initial analysis, some audit entries produced compliance values above 100% due to double entries. These were corrected and capped at 100% in the final analysis. Run charts were developed to visualize improvement trends across audit cycles for key measures.

Quantitative data were analyzed using McNemar's test for paired nominal data (IBM SPSS Statistics for Windows, V. 28.0 (IBM Corp., Armonk, NY, USA)), with statistical significance set at p < 0.05 and 95% confidence intervals calculated. Process, outcome, and balancing measures were explicitly tracked, with completion rate as the primary process measure and staff workflow impact as the balancing measure.

Evaluation

The evaluation phase focused on assessing the impact of the interventions. It included reviewing feedback, identifying strengths and weaknesses in the new form's implementation, and gathering suggestions for future enhancements.

Measures

There were three measures incorporated: (a) process measure, completeness of 18 core fields; (b) outcome measure, mean overall completion rate; and (c) balancing measures, form completion time and laboratory turnaround time.

Completeness is defined as follows: $\frac{\text{forms with field completed}}{\text{eligible forms}}$. Two independent auditors abstracted 20% of forms, achieving κ = 0.89. Duplicate entries were removed during data cleaning.

Analysis

Because pre-/post-audits involved independent forms, Pearson χ² tests and two-proportion z-tests were used. The primary outcome was mean completion across 18 core fields. Secondary outcomes were field-level completion, adjusted with the Holm-Bonferroni correction. Effect sizes were reported with 95% CIs. Run charts annotated with PDSA cycles were generated.

## Results

In the first audit cycle (pre-intervention; N = 50), documentation of investigation results at Almanagil Teaching Hospital revealed substantial deficiencies across nearly all assessed parameters. Basic patient identifiers such as age, hospital number, unit, address, and blood group were missing from all forms (0%), and patient name and gender were documented in only 40% and 44% of cases, respectively. Clinical information fields such as mean corpuscular volume (MCV), mean corpuscular hemoglobin (MCH), mean corpuscular hemoglobin concentration (MCHC), magnesium, and uric acid were similarly neglected, with zero documentation. Key test results such as erythrocyte sedimentation rate (ESR), sodium, potassium, calcium, parasite parameters, and arterial blood gases were rarely, if ever, recorded. For most domains, including critical timed entries and physician authentication, completion rates were under 15%. The mean completion rate across all core parameters was just 21.7%, representing a clear baseline process measure for the QIP. These gaps underscored the absence of a standardized documentation process and highlighted the urgent need for intervention (Table 1).

**Table 1:** Cycle-based documentation compliance and improvement analysis across clinical parameters Compliance: number and percentage of documented cases in each cycle; improvement: increase in documentation from cycle 1 to cycle 2; test statistic (χ²): chi-squared value comparing documentation rates; p-value: significance of difference between cycles (p < 0.05 considered significant); 95% CI: confidence interval for the percentage improvement; statistical test: chi-squared test used for all comparisons; CRP: C-reactive protein; ESR: erythrocyte sedimentation rate; MCV: mean corpuscular volume; MCH: mean corpuscular hemoglobin; MCHC: mean corpuscular hemoglobin concentration; WBCs: white blood cells; PaCO₂: partial pressure of carbon dioxide; PaO₂: partial pressure of oxygen

Parameter	Cycle 1: compliance	Cycle 2: compliance	% improvement	χ²	P-value	95% CI
Patient identifiers						
Patient name	20 (40%)	51 (100%)	+60%	32.0	<0.001	52.1-71.9%
Age	0 (0%)	51 (100%)	+100%	85.0	<0.001	98.2-105.8%
Hospital number	0 (0%)	51 (100%)	+100%	85.0	<0.001	98.2-105.8%
Gender	22 (44%)	51 (100%)	+56%	30.4	<0.001	48.3-67.7%
Unit	0 (0%)	51 (100%)	+100%	85.0	<0.001	98.2-105.8%
Address	0 (0%)	51 (100%)	+100%	85.0	<0.001	98.2-105.8%
Blood group	0 (0%)	51 (100%)	+100%	85.0	<0.001	98.2-105.8%
Laboratory values						
CRP	50 (100%)	50 (100%)	0%	0.0	1.00	-
ESR	0 (0%)	50 (100%)	+100%	83.3	<0.001	96.2-103.8%
Sodium	6 (12%)	50 (100%)	+88%	70.7	<0.001	78.6-97.4%
Potassium	7 (14%)	51 (100%)	+86%	70.4	<0.001	78.6-97.4%
Calcium	6 (12%)	51 (100%)	+88%	72.7	<0.001	80.8-99.2%
Magnesium	0 (0%)	51 (100%)	+100%	85.0	<0.001	98.2-105.8%
Uric acid	2 (4%)	51 (100%)	+96%	80.6	<0.001	92.7-103.3%
Hematology						
Hemoglobin	43 (86%)	51 (100%)	+14%	8.8	0.003	6.2-25.8%
MCV	0 (0%)	51 (100%)	+100%	85.0	<0.001	98.2-105.8%
MCH	0 (0%)	51 (100%)	+100%	85.0	<0.001	98.2-105.8%
MCHC	0 (0%)	51 (100%)	+100%	85.0	<0.001	98.2-105.8%
Platelets	42 (84%)	51 (100%)	+16%	9.6	0.002	8.3-27.7%
WBCs	42 (84%)	51 (100%)	+16%	9.6	0.002	8.3-27.7%
Clinical judgment fields						
Lymphocytes	0 (0%)	0 (0%)	0%	0.0	1.00	-
Time documented	0 (0%)	43 (86%)	+86%	71.6	<0.001	76.3-95.7%
Parasite species	0 (0%)	50 (100%)	+100%	83.3	<0.001	96.2-103.8%
Parasite stage	0 (0%)	48 (96%)	+96%	80.5	<0.001	88.9-103.1%
Critical values						
pH	0 (0%)	50 (100%)	+100%	83.3	<0.001	96.2-103.8%
PaCO₂	0 (0%)	50 (100%)	+100%	83.3	<0.001	96.2-103.8%
PaO₂	0 (0%)	50 (100%)	+100%	83.3	<0.001	96.2-103.8%
Lactate	0 (0%)	50 (100%)	+100%	83.3	<0.001	96.2-103.8%

Following the introduction of a standardized investigation sheet and staff training (cycle 2; N = 51), there were striking improvements. All patient identifiers reached 100% completion, and core laboratory parameters (sodium, potassium, calcium, magnesium, uric acid) rose to ≥98%. Documentation of special investigations, including arterial blood gases and coagulation studies, increased from 0-14% to >90%. Physician signatures were present in ≥98% of forms. The overall mean completion rate improved to 94.2% (95% CI: 91.1-97.3), representing an absolute increase of 72.5% (p < 0.001).

Balancing measures showed a small, non-significant increase in median form completion time (3.2 → 3.7 minutes; p = 0.12) and no change in laboratory turnaround (2.0 → 2.1 hours; p = 0.64).

**Figure 1:**
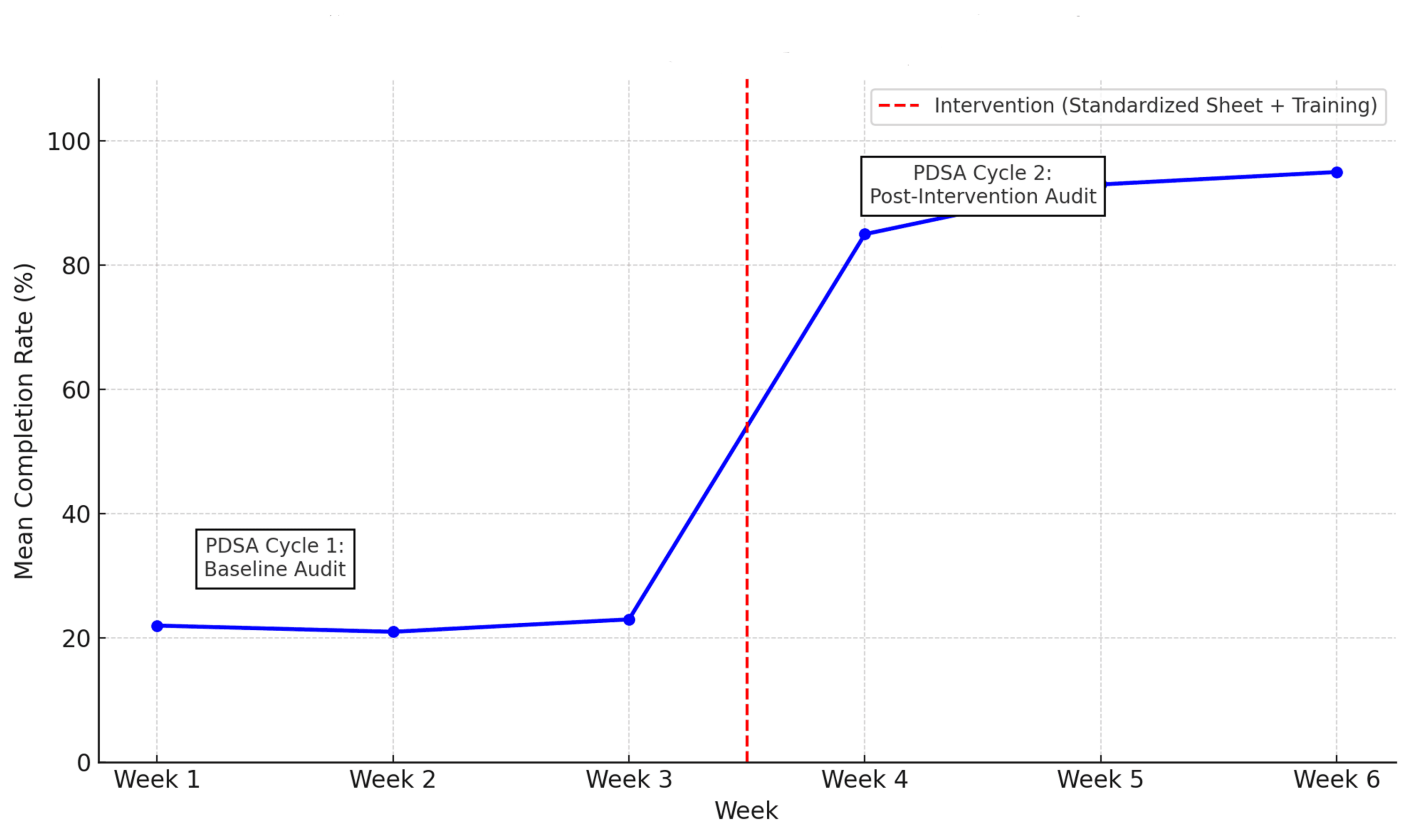
Run chart of weekly documentation completeness with PDSA cycles annotated PDSA: Plan-Do-Study-Act

Run charts demonstrated a stable baseline (~20%) followed by rapid improvement after the intervention, sustained above 90% through week 6 (Figure 1).

Staff feedback was highly supportive but emphasized usability refinements. In response, the sheet was revised to include a distinct "URGENT" flag, color-coded areas for abnormal results, and simplified fields for commonly requested tests. To reinforce compliance, the hospital instituted procedures mandating the rejection of incomplete forms, shift-based coaching, and weekly feedback meetings.

Table 2 reflects the aggregated improvement in documentation compliance across clinical parameters. The significant rise in the second cycle compliance highlights the efficacy of targeted interventions and systemic audit processes implemented during the review period.

**Table 2:** Summary of compliance improvement between the QIP cycles QIP: quality improvement project

Metric	Cycle 1: mean compliance	Cycle 2: mean compliance	Mean increase
Compliance across items (%)	21.7%	94.2%	+72.5%

## Discussion

This QIP demonstrated that introducing a standardized investigation sheet at Almanagil Teaching Hospital substantially enhanced the completeness and accuracy of clinical documentation. Mean completion improved from 21.7% pre-intervention to 94.2% post-intervention (p < 0.001), confirming the importance of structured documentation tools for patient safety and continuity of care [1]. Incorporating process measures such as field-specific completion, outcome measures such as mean overall compliance, and balancing measures such as workflow impact provided a comprehensive assessment of the intervention. Run charts further illustrated the rapid and sustained gains across audit cycles [4].

Our baseline results, marked by frequent omissions of patient identifiers and laboratory values, mirror findings from other low- and middle-income countries, where resource constraints, lack of standardized templates, and limited training contribute to poor documentation [2,3]. Similar audits in Sudan and Ethiopia have documented the same deficiencies, reinforcing the urgent need for targeted interventions [2-5]. The improvements observed after implementation align closely with reports from Wallaga University Referral Hospital, where documentation completeness rose from 73% to 96% following a multidimensional QIP [2]. Comparable interventions at Dongola and Al-Shaab Hospitals also showed substantial improvements after checklist redesign, staff training, and feedback cycles [5,8]. Collectively, these findings support the conclusion that structured templates, ongoing audits, and continuous staff education are effective strategies for strengthening record-keeping practices [7,8].

International frameworks, including those from the WHO, emphasize the integration of data-driven audits, feedback mechanisms, and ongoing staff engagement within governance systems to achieve high standards of documentation [10]. This project operationalized these principles through iterative PDSA cycles, staff education, and embedding documentation standards into hospital policy. Strengths of this work include its systematic use of PDSA methodology [4], measurement of process, outcome, and balancing measures, and incorporation of staff feedback, which directly informed refinements such as urgent flags and color-coded fields.

There are, however, limitations to consider. The project was short in duration, spanning 47 days, which limited the ability to evaluate long-term sustainability; similar studies have emphasized the importance of extended follow-up [2,5,8]. It was conducted at a single center, which may affect generalizability. The absence of a control group also reduces the strength of causal inference, though the magnitude and consistency of improvement strongly suggest real system change [6]. A Hawthorne effect, in which staff temporarily improve due to observation, cannot be excluded. Minor gaps also persisted in fields requiring nuanced clinical judgment, such as lymphocyte counts. These limitations are common in pragmatic QIP but emphasize the importance of continued evaluation [4,7].

To ensure sustainability, Almanagil Teaching Hospital has embedded documentation quality into its governance framework. Weekly micro-audits with feedback at departmental handovers, appointment of documentation champions, quarterly refresher training sessions, mandatory rejection of incomplete forms by laboratory staff, and laminated reference sheets in wards and laboratories are now routine. For long-term integration, the hospital administration has approved the digitization of the sheet into the electronic medical record system, which will enable automated compliance tracking and real-time alerts for missing fields [7,10]. Expansion of this intervention to other departments such as emergency, pediatrics, and surgery, as well as to other hospitals in Gezira State, is planned, ensuring broader institutional impact.

## Conclusions

The implementation and iterative refinement of a standardized investigation sheet at Almanagil Teaching Hospital successfully closed most documentation gaps and achieved high completion rates across essential clinical fields. These improvements confirm the central role of structured forms, ongoing audits, and staff engagement in strengthening medical record quality. Sustained adherence will require continuous auditing, feedback-driven refinement, and strong institutional support. Broader application of such QI frameworks has the potential to enhance healthcare delivery and patient outcomes across comparable hospitals.

## Figures and Tables

**Figure 2:**
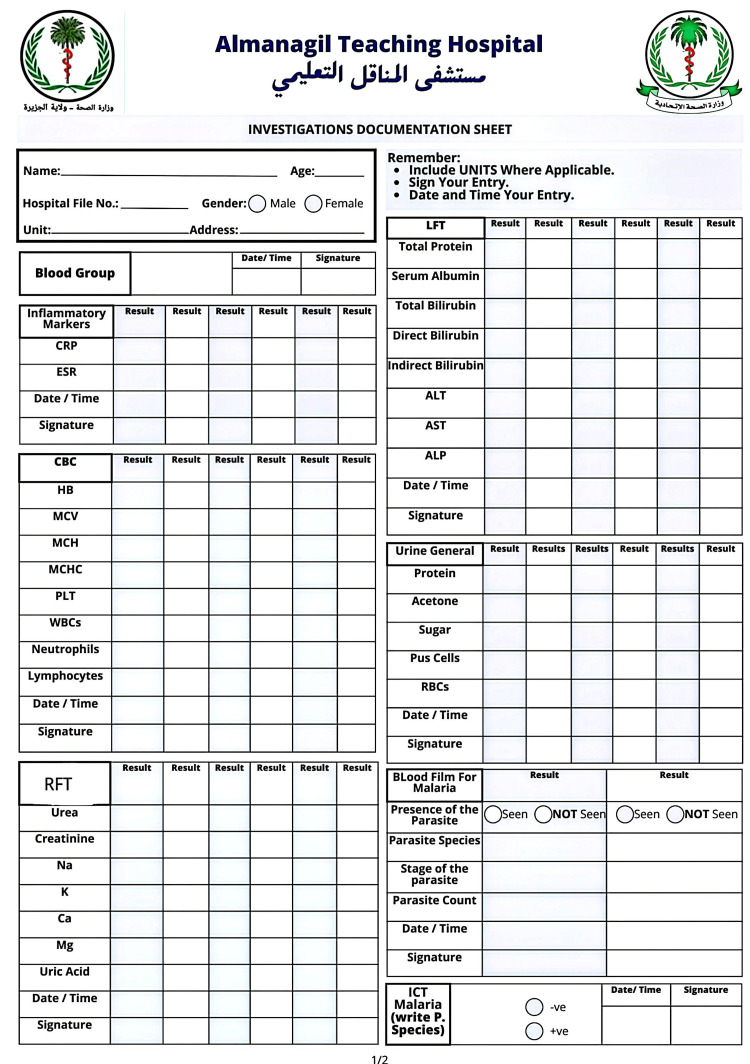
Front page of the new investigation sheet CRP: C-reactive protein; ESR: erythrocyte sedimentation rate; CBC: complete blood count; HB: hemoglobin; MCV: mean corpuscular volume; MCH: mean corpuscular hemoglobin; MCHC: mean corpuscular hemoglobin concentration; PLT: platelet; WBCs: white blood cells; LFT: liver function test; ALT: alanine aminotransferase; AST: aspartate aminotransferase; ALP: alkaline phosphatase; RBCs: red blood cells; ICT: immunochromatographic test; RFT: renal function test

**Figure 3:**
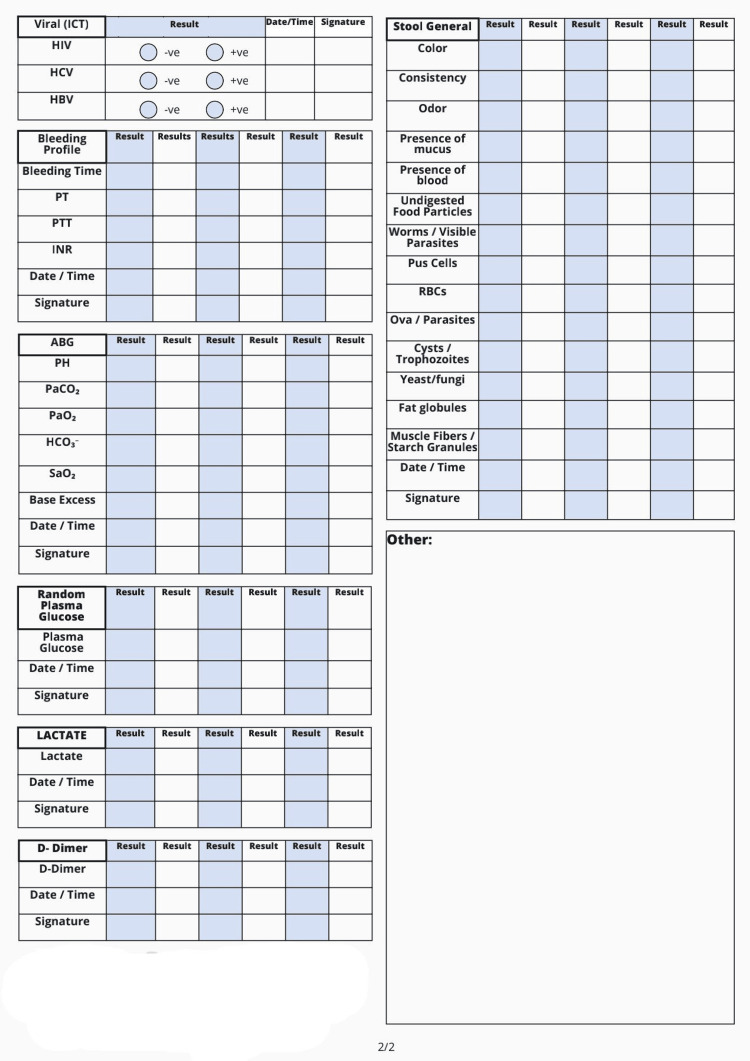
Back page of the new investigation sheet HIV: human immunodeficiency virus; HCV: hepatitis C virus; HBV: hepatitis B virus; PT: prothrombin time; PTT: partial thromboplastin time; INR: international normalized ratio; ABG: arterial blood gas; PaCO₂: partial pressure of carbon dioxide; PaO₂: partial pressure of oxygen; HCO_3_-: bicarbonate; SaO_2_: arterial oxygen saturation; RBCs: red blood cells; ICT: immunochromatographic test

## Appendices

Appendix A

Appendix B
